# Visual scanning of social stimuli in preterm and autism spectrum disorder children

**DOI:** 10.1590/1984-0462/2024/42/2023017

**Published:** 2024-05-06

**Authors:** Vivian Renne Gerber Lederman, Ana Lucia Goulart, Juliana Gioia Negrão, José Salomão Schwartzman

**Affiliations:** a Universidade Presbiteriana Mackenzie, São Paulo, SP, Brazil.; b Universidade Federal de São Paulo, São Paulo, SP, Brazil.

**Keywords:** Autism, Autism spectrum disorder, Eye-tracking technology, Infant, premature, Social visual engagement, Autismo, Transtorno do espectro do autismo, Rastreamento ocular, Prematuridade, Engajamento social visual

## Abstract

**Objective:**

To evaluate the pattern of eye-gaze of preterm (PT), autism spectrum disorder (ASD) and neurotypical (Ty) children.

**Methods:**

A cross-sectional study with eight preterm (born with ≤2000 g weight), nine ASD and five Ty male children, between six and nine years old, was performed. The eye gaze was evaluated presenting a board with a couple in social interaction, and a video with four children playing with blocks, projected in a screen computer, successively, evaluating the time that the children looked at each stimulus.

**Results:**

Although all the groups focus on the central social figure with no significant differences, ASD presented significant differences in time fixation of the objects (p=0.021), while premature children fixated more time in the central social interaction than in the whole scene than typical children.

**Conclusions:**

Although this study found noteworthy differences in the eye-gaze patterns among the three groups, additional research with a more extensive participant pool is necessary to validate these preliminary results.

## RESUMO

**Objetivo:**

Avaliar o padrão de fixação visual de crianças nascidas prematuras (PT), com transtorno do espectro do autismo (TEA/ASD) e neurotípicas (Ty).

**Métodos:**

Estudo transversal incluindo oito meninos prematuros (nascidos com peso ≤2000 g), nove TEA e cinco Ty, com idade entre 6 e 9 anos. A varredura visual foi avaliada apresentando-se imagem de casal em interação social e vídeo com crianças brincando. A figura e o vídeo foram projetados em tela de computador, sucessivamente, sendo avaliado o tempo de fixação para cada estímulo.

**Resultados:**

Apesar de os três grupos de crianças focarem o olhar na figura social central sem diferenças significativas, crianças com TEA apresentaram diferenças significativas no tempo de fixação em objetos (p=0,021), enquanto prematuros fixaram o olhar mais tempo na interação social central do que na cena como um todo.

**Conclusões:**

Embora tenham sido notadas algumas diferenças significativas nos padrões de olhar para os três grupos, é necessário realizar mais pesquisas com uma amostra maior para confirmar esses resultados iniciais.

## INTRODUCTION

Neurotypical (TY) newborns (those whose neurological development follows the expected pattern for their age, without presenting significant neurological conditions or disorders) show visual preferences for social stimuli from the very first days of their lives.^1^ On the other hand, full-term children latter diagnosis as having autism spectrum disorder (ASD) show social figure eye gaze decline between two and six months of age, which does not occur in Ty term children. The lack of interest in social figures could be a sign of social impairment and may be identified from the first child life months through eye tracking. The eye tracking equipment records the visual scanning of the child and helps to determine how a child explore the ambience, providing important contributions to the comprehension of social gaze and social interactions.^2^

Few studies have analyzed premature children’s gaze patterns, although they are a well-known group of risk for several behavioral impairments.^3^ The first visual scanning studies with preterm (PT) babies were not conclusive as to whether they have a similar pattern of visual scanning as neurotypical children,^4^ or present impairments due to prematurity or signs of ASD.^5^

The present work hypothesizes that PT children may have a different pattern of eye-gaze from ASD and Ty groups, if evaluated later in development, and those patterns should be better understood.

## METHOD

This brief report is an exploratory cross-sectional study that was conducted after approval by the ethics committees of Universidade Presbiteriana Mackenzie (protocol #2.886.398) and after all parents or guardians and children had signed a consent form. A convenience sample including eight PT with birth weight ≤2000 g, nine children with ASD diagnosis, according to DSM-5,^6^ and five Ty children, born at term and with no ASD diagnosis, was evaluated. The inclusion criteria were boys between six and nine years old and a minimum IQ>70, measured through Wechsler Abbreviated Scale Intelligence.^7^ The exclusion criteria were genetic syndrome and/or presence of a major motor, visual, or hearing impairment.

**Figure 1:**
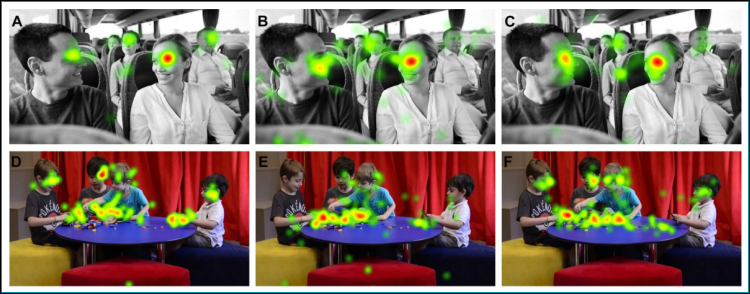
Eye-tracking results.

Visual scanning was evaluated with eye-tracking Tobii Pro Lab X3-120 (Tobiipro AB, Stockholm, Sweden). This equipment is able to monitor and record the eye gaze of a person in a screen, recording not only where the person is looking, but also what the pattern of the gaze and eye fixation time in the screen is. In this work, as stimuli, one board and one video were used, with 5 seconds of exposure each one (Figure 1). The board (Figure 1 A, B and C) showed a couple in social interaction on a bus, and the video (Figure 1 D, E and F) showed three children playing with blocks and a fourth child by the side. Only one session of one hour was performed in the presence of parents or guardians. All the participants were able to focus appropriately on the equipment, and the eye tracker was capable of tracking more than 70% of the scanning time. Time of fixation was elected as a parameter, following previous research.^4,5^ A single trained researcher extracted the data from the equipment. Only one session of one hour was performed in the presence of parents or guardians. The percentage of eye gaze of the infants on social first and second stimuli were compared using the Wilcoxon test and variance analysis (ANOVA), considering a significant difference, with p<0.05.

## RESULTS

Social economics measures of the PT group were lower than Ty and ASD groups (p=0.20). Intelligence quotient (IQ) measures of the Ty group were superior to PT and ASD groups (p=0.05) (Table 1).

**Table 1:** Fixation duration of visual scanning.

					Mean	Median	SD	n	95%CI	p-value
Figure 1 – A Couple on a bus		Women’s eyes	Typical		0.73	0.67	0.54	5	0.48	0.918
			Premature		0.69	0.66	0.45	10	0.28	
			ASD		0.62	0.90	0.57	9	0.37	
		Man’s eyes	Typical		0.52	0.62	0.35	5	0.31	0.021
			Premature		0.55	0.45	0.51	10	0.32	
			ASD		0.06	0.00	0.11	9	0.07	
		Bus background	Typical		1.60	1.49	0.41	5	0.36	0.021
			Premature		0.89	0.77	0.46	10	0.29	
			ASD		1.31	1.44	0.46	9	0.30	
Video – Children’s interaction		Child 4	Typical		0.47	0.38	0.31	5	0.27	0.042
			Premature		0.18	0.04	0.25	10	0.15	
			ASD		0.11	0.00	0.21	9	0.14	
		Blocks	Typical		0.16	0.20	0.16	5	0.14	0.002
			Premature		1.04	1.12	0.67	10	0.41	
			ASD		1.96	2.04	1.12	9	0.73	
		Child 1	Typical		0.32	0.12	0.44	5	0.39	0.084
			Premature		0.20	0.06	0.26	10	0.16	
			ASD		0.00	0.00	0.00	9	- x -	
		Child 2	Typical		0.62	0.71	0.51	5	0.45	0.089
			Premature		0.41	0.27	0.47	10	0.29	
			ASD		0.12	0.00	0.20	9	0.13	
		Child 3	Typical		0.21	0.22	0.22	5	0.19	0.241
			Premature		0.43	0.27	0.55	10	0.34	
			ASD		0.13	0.16	0.13	9	0.09	
Differences between groups										
		Typical-premature		Typical-ASD		Premature-ASD				
Figure 1 - A couple on a bus	Man’s eyes	0.991		0.089		0.024				
	Bus background	0.022		0.486		0.126				
Video - Children’s interaction	Child 4	0.095		0.038		0.821				
	Blocks	0.151		0.002		0.056				

SD: standard deviation; 95%CI: 95% confidence interval; ASD: autism spectrum disorder.

Regarding the eye-tracking on the board (Figure 1A, B and C), where the central social figure is a woman, and a second, lateral figure is a man, the three groups did not present significant differences in eye-gaze towards the central social stimulus (woman) (p=0.918), but there were significant differences towards the second social stimulus (man) (p=0.21). The man’s eyes were less scanned by ASD than PT (ASD 0.06 sec vs. PT 0.89 sec, p= 0.024) and tended to be less scanned by ASD than Ty (ASD 0.06 vs. Ty 0.52 sec, p=0.089). The PT and Ty groups did not show significant differences (p=0.991). Fixation time on the back of social interaction (bus) presented a significant difference of eye-gaze between the three groups (p=0,021), with the PT group presenting less fixation than Ty (0.89 sec vs. 1.60 sec, p=0,022). The PT group seemed to fix their attention on the social interaction, not screening the whole scene, while ASD seemed to gaze from the central social figure to non-social aspects.

In the video (Figure 1D, E and F), children 1, 2 and 3 (central social figure) played with blocks and child 4 did not participate in the social interaction. There were significant differences between the groups in the blocks’ fixation time (non-social stimulus) (p=0.02), ASD being superior to PT (p=0.056) and Ty (p= 0.02). Regarding the social stimuli (children faces), there was no significant difference among the three groups considering the fixation time on Child 3 (p=0.241), the central social figure. ASD did not present fixation time on Child 1, and Ty tended to fix more than PT (PT 0.20 sec vs. Ty 0.32 sec, p=0.084). This tendency could be also observed regarding Child 2 (ASD 0.12 sec vs. PT 0.41 sec vs. Ty 0.62, p=0.089). Although the central social figure seemed to have been scanned by the three groups, secondary social figures tended to be less gazed by PT and ASD groups. Eye-tracking of Child 4 showed significant differences between the groups (p=0.042). ASD time fixation was inferior than Ty (ASD 0.11 sec vs. Ty 0.47 sec, p=0.038) and PT fixation time was inferior to Ty group (PT 0.18 sec vs. Ty 0.47 sec, p=0.095).

## DISCUSSION

Our preliminary eye-gaze study comparing ASD and premature children showed that although the groups appear to primarily focus on social figures with no significant differences when compared to Ty children, PT and ASD children tend to fixate less on secondary social stimuli. ASD showed gaze on objects present at the scene, while premature fixed their gaze on part of the social interaction, but eventually not on whole interaction as Ty children.

An adequate visual scan of a social situation is important to comprehend how to behave and give a suitable response.^8^ The lack of a complete visual scan of a social situation could lead to behavioral difficulties. Although few are the studies with eye-tracking of premature babies, they can perform visual scanning from six months of corrected age, being a helpful tool to elucidate ASD signs or neurodevelopmental delays.^4^ Premature babies present a differential pattern of eye-gaze in the first month of life,^9,10^ and our results highlight the possibility of maintenance of a differential pattern through childhood or even later in life. As limitations of this study, we include the small and convenient sample, as well as the lack of IQ and social economics matching among the groups. This study specifically focuses on children aged 6–8, and it’s possible that the findings may not generalize to other age groups. We conclude that differences in the pattern of eye-gaze between Ty, PT and ASD children can be present, and those differences should be investigated in additional research with a larger sample.

## Data Availability

The database that originated the article is available with the corresponding author.
